# Efficacy and adverse reaction management of oncolytic viral intervention combined with chemotherapy in patients with liver metastasis of gastrointestinal malignancy

**DOI:** 10.3389/fonc.2023.1159802

**Published:** 2023-05-01

**Authors:** Jie Zhang, Qianyun He, Dongliang Mao, Chen Wang, Lei Huang, Mei Wang, Jun Zhang

**Affiliations:** ^1^ Department of Nursing, Ruijin Hospital, Shanghai Jiao Tong University School of Medicine, Shanghai, China; ^2^ Department of Oncology, Ruijin Hospital, Shanghai Jiao Tong University School of Medicine, Shanghai, China; ^3^ Medical Center on Aging of Ruijin Hospital (MCARJH), Shanghai Jiao Tong University, School of Medicine, Shanghai, China

**Keywords:** gastrointestinal malignancy, liver metastasis, oncolytic virus, management of adverse reactions, nursing procedures, interventional radiology introduction

## Abstract

**Background:**

The liver is a key target organ for colorectal and gastric cancer metastasis. One of the challenges in the treatment of colorectal and gastric cancers is the management of liver metastasis. This study aimed to investigate the efficacy, adverse effects, and coping strategies of oncolytic virus injection in patients with liver metastases of gastrointestinal malignancies.

**Methods:**

We prospectively analyzed patients treated at Ruijin Hospital affiliated with Shanghai Jiao Tong University School of Medicine from June 2021 to October 2022. 47 patients with gastrointestinal cancer liver metastasis were included in the study. The data, including clinical manifestations, imaging, tumor markers, postoperative adverse reactions, psychological intervention, dietary guidance, and adverse reaction management were evaluated.

**Results:**

Oncolytic virus injection was successful in all patients, and no drug injection-related deaths occurred. The adverse effects, such as fever, pain, bone marrow suppression, nausea, and vomiting, were mild and resolved subsequently. Based on the comprehensive intervention of nursing procedures, the postoperative adverse reactions of patients were effectively alleviated and treated. None of the 47 patients had any puncture point infections, and the pain caused by the invasive operation was relieved quickly. After 2 courses of oncolytic virus injection, postoperative liver MRI showed 5 partial remissions, 30 stable diseases, and 12 progressive diseases in target organs.

**Conclusion:**

Interventions based on nursing procedures can ensure the smooth treatment of recombinant human adenovirus type 5 in patients with liver metastases of gastrointestinal malignant tumors. This is of great importance for clinical treatment and significantly reduces patient complications and improves the patient’s quality of life.

## Introduction

Liver metastasis is the leading cause of death in patients with colorectal and gastric cancer (1), with the 5-year survival rate being below 5%. Colorectal and gastric cancers are commonly associated with liver metastasis (2), with approximately 15–25% of colorectal and gastric cancer patients having liver metastasis at diagnosis. Moreover, 15–25% of patients who underwent radical resection of primary colorectal and gastric cancer lesions will have liver metastasis (3), with the vast majority (80-90%) of liver metastases being impossible to obtain by initial radical resection (4).

Oncolytic viruses are a class of natural or recombinant viruses that selectively infect and kill tumor cells without damaging normal cells (5, 6). Compared to traditional immunotherapies (7), oncolytic virotherapy has advantages such as superior tumor targeting, minimal adverse reactions, multiple tumor-killing routes, and is less prone to drug resistance (8). Several clinical studies have shown that oncolytic viruses can bring clinical benefits to patients with different cancer types and stages of progression (9). It has also been shown that oncolytic viruses can even provide clinical benefits for metastatic and incurable tumors (10). More importantly, oncolytic viruses show synergistic effects in combination with traditional anti-cancer regimens such as chemotherapy, radiotherapy, and immunotherapy (8), which can potentiate tumors that respond poorly to immunotherapy drugs such as immune checkpoint inhibitors (11).

## Materials and methods

### Ethical approval of the study protocol

The present study protocol was approved by the Ethical Investigation Committee of Ruijin Hospital, affiliated with the School of Medicine, Shanghai Jiaotong University, China. Written informed consent was obtained from every patient. Questionnaires were filled to collect and analyze the applicable clinical data.

### Data source

We prospectively analyzed the clinical data of patients with liver metastasis of gastrointestinal malignant tumors who received oncolytic virus injections at Ruijin Hospital, School of Medicine, China from June 2021 to October 2022.

### Inclusion and exclusion criteria

The inclusion criteria were as follows: (1) patients need sign an informed consent to participate in the study; (2) the injectable lesions in the liver meet the requirements of the RECIST1.1 for measurable target lesion; (3) the longest diameter of the liver lesions is no greater than 100 mm, and diameter of the injection lesion is between 10 mm and 80 mm; and (4) current physical condition and following treatment plan were confirmed consistent to the protocol by investigator. The exclusion criteria were as follows: (1) injectable lesions had received ablation, intervention, offshore knife, and other local treatments before; (2) oncolytic virus or similar drug (such as T-VEC) was performed before; (3) received antiviral treatment, such as acyclovir, ganciclovir, and alsidine, within 4 weeks before the first dose of the trial treatment; (4) pregnant or breastfeeding women or those ready to conceive or feed during the study; and (5) combined with other diseases that could influence the study.

**Figure 1:**
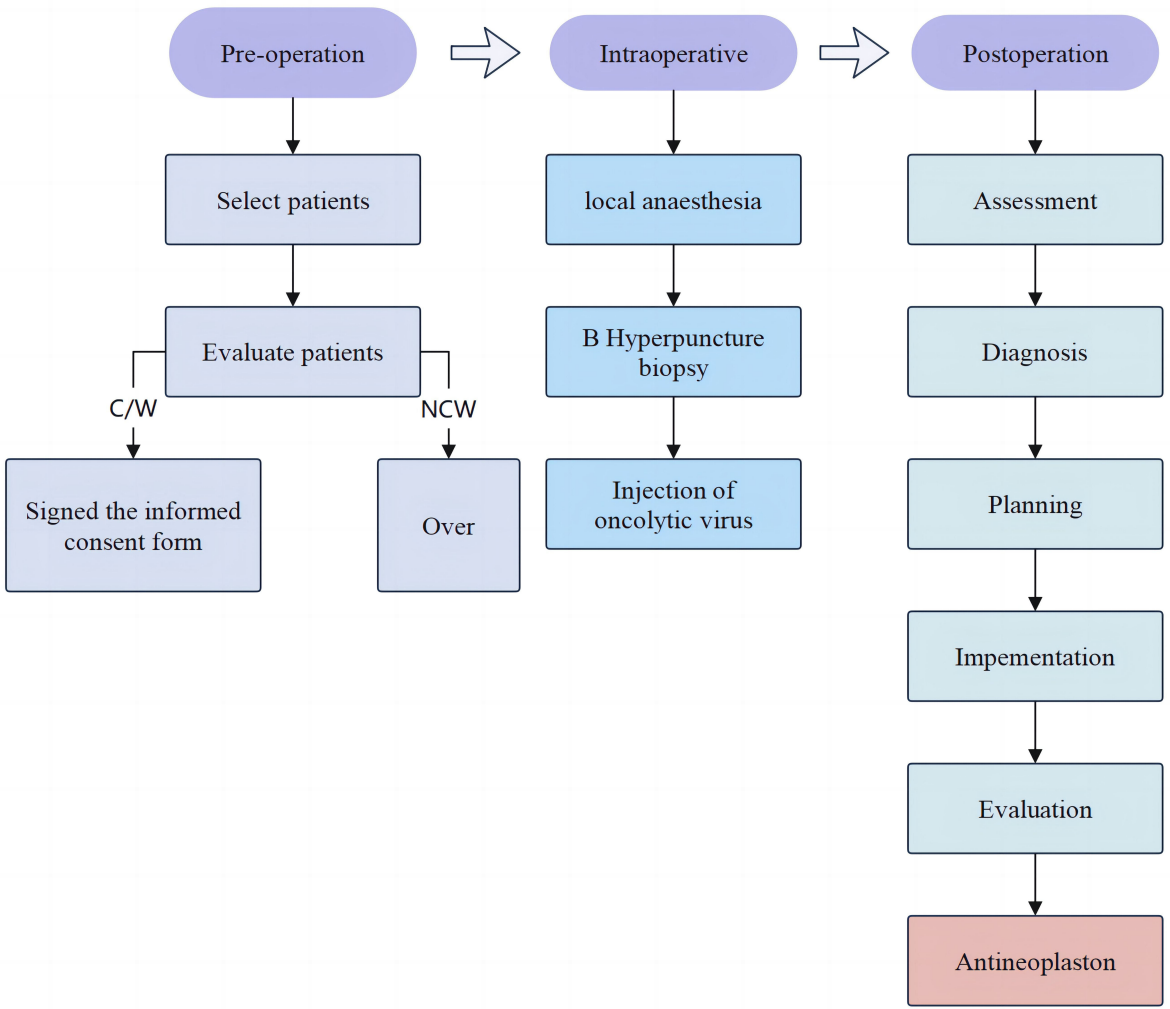
Flow diagram of the study process.

The attending performed the oncolytic interventional surgery and managed the adverse reactions before and after surgery along with the nurses according to the nursing procedures. The nursing procedure involves evaluation, diagnosis, planning, implementation, and evaluation (**Figure 1**). Nursing process is a theoretical and practical model that guides medical staff to implement planned, continuous and comprehensive nursing in a systematic way with the goal of satisfying the physical and mental needs of the nursing object and restoring or improving the health of the nursing object (12). Nursing Process is a planned, systematic and scientific method of work aimed at identifying and resolving clients’ reactions to existing or potential health problems (13, 14).

### Preoperative preparation

The patient’s allergy history was asked in detail, and the allergy was confirmed during the operation. Prior to the surgery, parameters such as tumor markers, coagulation function, blood routine, and liver function were checked. Further, the nurse instructed the patient to have a light diet the day before the surgery and also to fast after 10 p.m. Before the surgery, the patient’s vital signs were evaluated again, and the patient was transported to the B-ultrasound room in a flat car (15). Before interventional treatment, patients were encouraged to eat high-calorie, high-vitamin, high-protein food to correct malnutrition. If there is a lack of appetite, a small number of meals were advised along with a walk before the meals to increase appetite and to improve physical quality.

### Procedure

Before the procedure, target liver lesion biopsy under the guidance of B-ultrasound was performed. Recombinant human adenovirus 5 injection (Shanghai 3 D Biotechnology Co., LTD., trade name Ancore, approval number: s20060027), with the following specification, was used: 5.0 × 10^11^ vp/0.5ml/branch, milky white suspension, main component name: recombinant human adenovirus 5 particles with E1B-55 kD and E3 region gene fragment (78.3–85.8 mu). Usage: After diluting the recombinant human adenovirus 5 injection with normal saline, three injection points were selected according to the longest tumor diameter under the guidance of B-ultrasound. The injection dose is determined according to the tumor size: if the longest diameter of the injected lesion was between 10 mm and 40 mm, 2 doses were injected; if the longest diameter of the injected lesion was between > 40 mm and 80 mm, 4 doses were applied. The recommended injection time was one cycle every 14 days and a total of two cycles intratumorally.

### Postoperative care

Patients were given psychological support before and after the procedure. The patients were guided after each intervention for both physical and mental relaxation (16). For this, the patients were asked to close their eyes, meditate, keep their palms by their bodies, breathe gently for 3–4 minutes, and slowly open their eyes and meditate for 30 min each time. This makes the patient relax each group of muscles and extend this feeling to the whole body. If the patient has severe anxiety, the patient may allow family members to accompany them for mental and emotional support (17).

After 4h post-infusion, the patient was allowed to consume a small amount of a light diet, followed by a high-calorie, high-vitamin, high-protein, warm, and cool semi-liquid diet the second day. Food that is fried, hard, boney, rough, prickly, or that requires chewing hard was avoided. Patients were encouraged to drink 2000–3000 ml of water or juice every day to reduce kidney complications (18).

### Disease observation

Routine blood work, liver function, DIC, tumor markers, and other tests, as well as a liver magnetic resonance examination, were completed 1 month after the operation to evaluate the curative effect. Vital signs such as blood pressure, heart rate, pulse, and respiration were closely monitored after the drug injection to assess the symptoms of pain and liver bleeding. If the patient appears pale, develops a cold sweat, exhibits a drop in pulse and blood pressure, or shows any other symptoms, timely symptomatic treatment is initiated (19).

Local observation and nursing of the puncture point are performed by covering with sterile gauze and then pressurizing with the abdominal belt. If necessary, the puncture point was pressed and the dressing was observed closely (20). After 24h, the dressing was removed, the puncture site was disinfected with iodophor, and the patient was checked for local bleeding and hematoma. The patient was allowed to rest slightly sideways for 4h, then advised to stay in bed for 24h. The patients were advised not to rub the puncture point for one week and to keep it dry and clean. If patients had a small amount of bleeding at the puncture point after the release of compression, further compression and covering with a sterile veil were necessary, until no bleeding was observed after veil removal 2 days later.

### Evaluation criteria for clinical efficacy and adverse reactions

RECIST 1.1 is an internationally recognized guideline for evaluating the efficacy of malignant tumors (21). It takes the maximum diameter of the tumor as the index of tumor measurement. Compared to the WHO standard, RECIST is more comprehensive and accurate. We proposed to measure the longest diameter of active tumors with enhanced CT or MRI, and arterial phase enhancement to evaluate the efficacy. This provides a more objective and realistic evaluation method for treatments that cause tumor necrosis rather than tumor shrinkage. A complete response (CR), partial response (PR), and whether the patient had stable disease (SD) or progression disease (PD) were used to assess the clinical efficacy of gastrointestinal oncology therapy. The CR + PR is defined as an objective response rate (ORR) and the disease control rate (DCR) is defined as the CR + PR + SD (22).

Carcinoembryonic antigen (CEA) is an important tumor marker, and the National Comprehensive Cancer Network (NCCN) (23) indicates that the changes in serum CEA levels are closely associated with the changes in the condition of tumor patients. In this study, the changes in CEA levels before and after the oncolytic virus infusion in patients with gastrointestinal malignancies were analyzed to determine the curative effect (24).

Liver function impairment classification (25), also known as Child-Pugh classification, is a commonly used standard for liver reserve function (26); it is mainly divided into A level (5–6 points), with normal liver function, B level (7–9 points), and C level (10–15 points), with liver function decompensation, including hepatic encephalopathy, ascites, serum bilirubin rise, serum albumin decline, and prothrombin time lengthen.

Evaluation and follow-up of liver function impairment: An increase in ALT exceeding the upper limit of normal (40 u/L) was defined as liver function impairment. Liver function impairment was graded according to the criteria of chemotherapy drugs and the NCI-CTC (3.0), which classified the liver function impairment into the following degrees: 0 degree indicates normal, and I degree is defined as aspartate aminotransferase/alanine aminotransferase (AST/ALT) 1.0 to 2.5 times the upper limit of normal [ULN] or total bilirubin (TB) 1.0 to 1.5 times ULN; II degree as AST/ALT 2.6 to 5 times ULN or TB 1.6 to 3 times ULN; III degree as AST/ALT 5.1 to 20 times ULN or TB 3.1 to 10 times ULN; IV degree as AST/ALT over 20 times ULN or TB over 10 times ULN (27).

The National Cancer Institute Common Toxicity Classification Criteria version 3.0 was used (28). Digital grading method: 0 indicates no pain; 1-3 mild pain, 4–6 moderate pain, and 7–10 severe pain.

### Statistical analyses

Data were analyzed using the SPSS 24.0 software (29), and the data were expressed as mean ± SD. Unpaired, student’s t-test was performed to check statistical significance; count data expressed by (%), P < 0.05 is considered statistically significant.

## Results

### General information

Patients were followed up for 6 months after the interventional therapy Forty-seven patients with recurrent liver metastasis of gastrointestinal malignant tumors admitted to our hospital from June 2021 to October 2022 met the diagnosis criteria for gastric adenocarcinoma or colorectal adenocarcinoma. A total of 47 patients, 34 (72%) males and 13 females (28%), with a mean age of 59.96 ± 13.43 years, comprised the study cohort. Of these 47 subjects, 22 patients had hypertension (47%), 8 patients had diabetes mellitus (17%), and 1 patient (2%) had a stroke. As per the tissue classification, 46 subjects had adenocarcinoma (97.9%). Further, 11 (23.4%) subjects were treated with chemotherapy plus immunotherapy, and 36 (76.6%) subjects received chemotherapy plus targeted therapy (**Table 1**).

**Table 1:** Patient characteristics (n = 47).

Variables	Categories	N (%) mean ± SD
Gender	male	34 (72%)
	female	13 (28%)
average age		59.96 ± 13.43
degree of education	Primary School	12 (26%)
	Middle School	10 (21%)
	High School	14 (21%)
	Junior college	5 (11%)
	undergraduate	5 (11%)
occupation	Retired	37 (79%)
	On the job	10 (21%)
Marital status	Married	43 (92%)
	Unmarried	4 (4%)
Basic disease	History of hypertension	22 (47%)
	History of diabetes	8 (17%)
	History of cerebral apoplexy	1 (2%)
Types of medication	History of intake of antihypertensive drug	18 (38%)
	History of intake of hypoglycemic drug	6 (13%)
	History of intake of analgesic	3 (6%)
	History of intake of psychotropic drug	1 (2%)
Tumor location	Colon cancer	25 (42.6%)
	Stomach cancer	8 (17.0%)
	Rectal cancer	14 (29.8%)
Clinical stage of tumor	IV	47 (100%)
Resectional surgery	Yes	36 (77%)
Tumor differentiation	Undivided type	5 (11%)
	adenocarcinoma	7 (15%)
	Medium-low differentiation	2 (4%)
Tumor histology	Adenocarcinoma	46 (97.9%)
	Squamous cell cancer	1 (2.1%)
therapy method	Chemotherapy + immunity	11 (17%)
	Chemotherapy + targeting	36 (77%)

### Evaluation of clinical efficacy

All 47 patients in this group received two injections.

**Figure 2:**
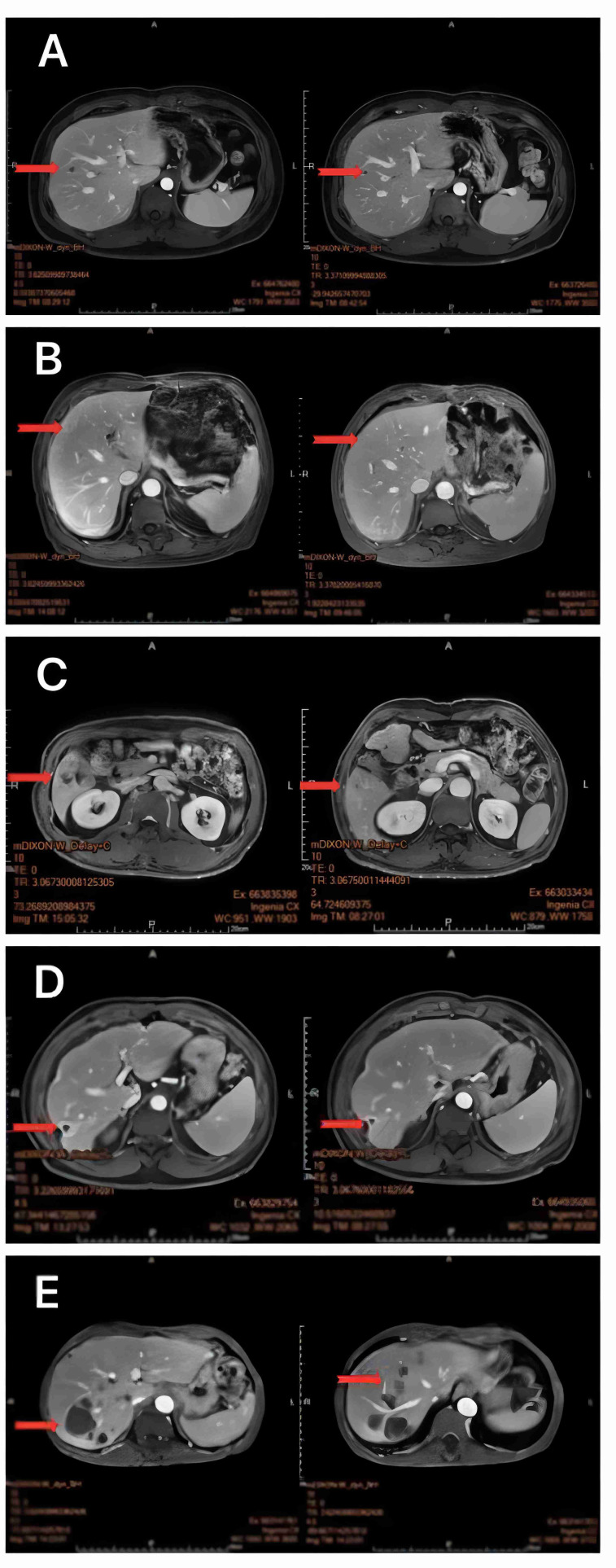
MRI of the liver 1 month after oncolytic virus injection showed a marked reduction in target organ shading **(A–E)**.

**Figure 3:**
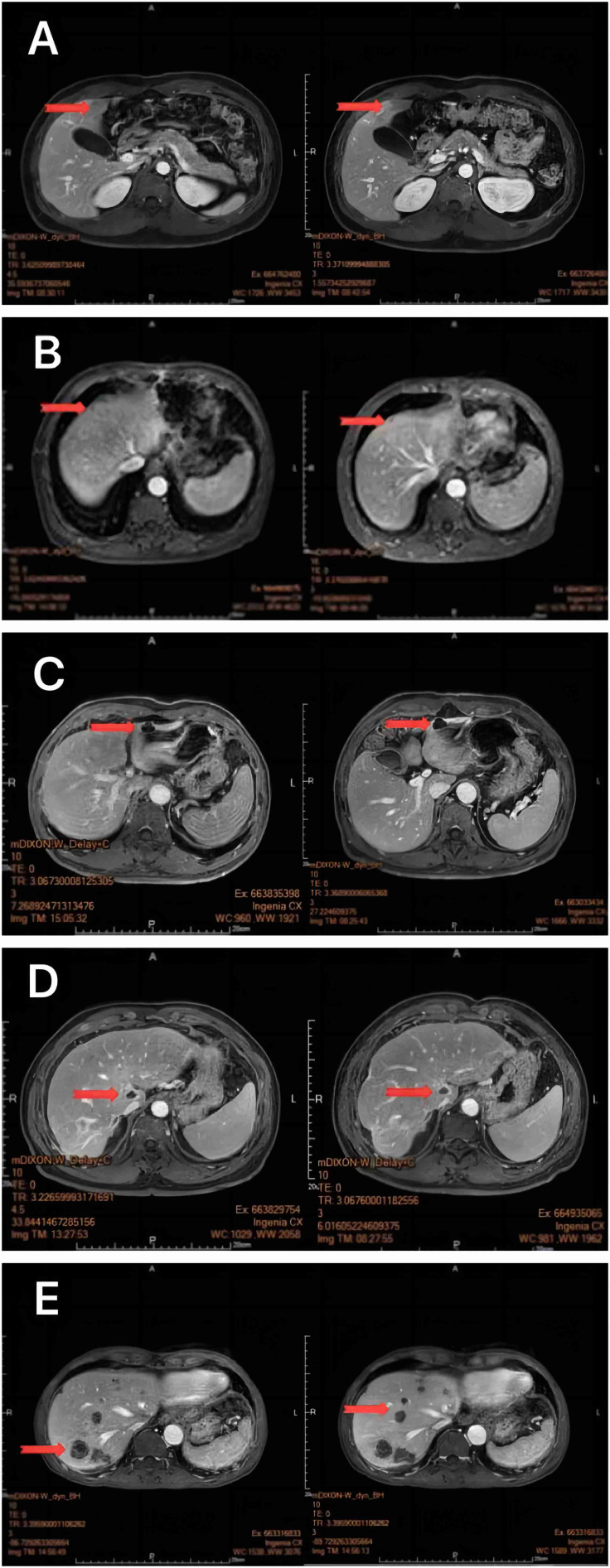
MRI of the liver 1 month after oncolytic virus injection showed a marked reduction in Non-Target Lesion **(A-E)**.

All 47 patients with liver metastasis of gastrointestinal malignant tumors were treated with oncolytic virus injection, followed by a liver MRI to evaluate the disease state. Our results suggest that 5 subjects were in remission, 30 had stable disease, and 12 subjects indicated a progressive disease. After symptomatic treatment and care for the adverse reactions, all patients were discharged smoothly (**Figures 2**, **3**; **Table 2**).

**Table 2:** Clinical efficacy after the oncolytic virus injection.

Variables	N (%)
SD	30 (63.8%)
PR	5 (10.6%)
PD	12 (25.5%)
Disease control rate	35 (74.5)

SD, Stable Disease.

PR, Partial Response.

PD, Progressive Disease.

Disease control rate = complete response + partial response + stable disease.

**Figure 4:**
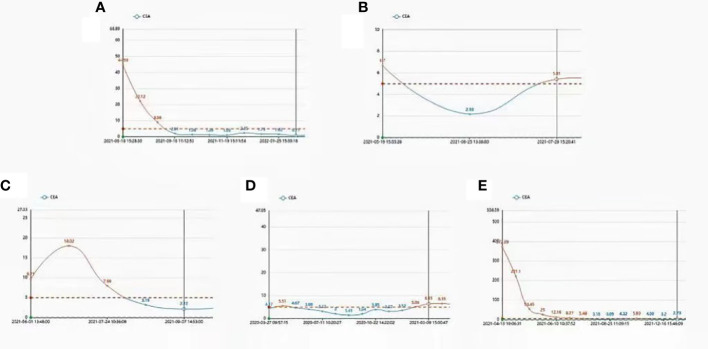
1 month, 3 months, 6 months, and CEA remained at a normal level **(A–E)**.

CEA is an important broad-spectrum tumor marker that was first identified in colon cancer and embryonic tissues. Although it cannot be used as a specific index for the diagnosis of a certain malignant tumor, it still has a certain value in the disease monitoring and efficacy evaluation of malignant tumors. To evaluate whether the oncolytic virus treatment has clinical benefit, we quantified CEA and found that CEA decreased after oncolytic virus injection compared to before injection and fluctuated within the normal range (**Figure 4**).

### Complications

Following the oncolytic virus injection, patients experienced fever and pain (**Table 3**). Five patients had nausea and vomiting (10.6%), 23 patients developed a low fever, 9 had a moderate fever, and 4 had a severe fever. Fever and the other common adverse effects were relieved after symptomatic treatment within a short time. Postoperative pain occurrence was also observed, with 18 patients indicating mild pain (38%) and 9 patients indicating moderate pain (19%). Furthermore, thrombocytopenia (9/47, 19.1%) and leukopenia (14/47, 29.8%) were observed in a small number of patients (**Table 3**). We also looked at the development of liver lesions before and after oncolytic virus injection (**Table 4**). Our results show that 39 patients exhibited preoperative grade Child A (83%) and 8 subjects had grade Child B (17%); 34 patients showed postoperative grade Child A (72%), and 13 subjects exhibited postoperative grade Child B (28%). There was no statistical difference between the two groups before and after surgery (P > 0.05).

**Table 3:** Adverse effects after oncolytic virus injection.

Variables	I	II	III
Fever	23 (49%)	9 (19%)	4 (9%)
Pain	18 (38%)	9 (19%)	–
Lowering of white blood cell count	9 (19%)	3 (6%)	2 (4%)
Thrombocytopenia;	7 (15%)	2 (4%)	0 (0%)

Low heat is 38°C; moderate heat is 38.1°C -38.9°C; and high heat is 39°C -40.9°C.

Pain score: 1-3 mild pain; 4 to 6 moderate pain; 7 to 10 severe pain.

Leukocyte reduction: less than 4.0 ^10^9/^L is leukopenia, mild: 3.5 ^ 10^9^/L, moderate above 3.0 ^ 10^9^/L, severe above 2.0 ^ 10^9^/L, less than 1.5 ^ 10^9^/L for deficiency.

Thrombocytopenia: Grade I: platelet count (75-100) ^ 10^9^/L, called mild platelet reduction, generally does not cause very obvious bleeding; Grade II: platelet count at (50-75) 10 ^ 9/L, the bleeding probability is small, patients can even tolerate minor surgical treatment; Grade III: platelet count (25-50) 10 ^ 9/L, easy to cause bleeding, such as bruises and bruises after a minor collision, gingival bleeding after brushing; Grade IV: platelet count below 25^10^9^/L, or severe thrombocytopenia, can cause spontaneous bleeding.

**Table 4:** Dynamic changes in liver function after oncolytic viral intervention.

Variables	Group	N (%)	0	I	II	III	IV
Preoperative	Child A	39 (83%)	26 (55%)	11 (23%)	2 (4%)	0 (0%)	0 (0%)
	Child B	8 (17%)	0 (0%)	4 (9%)	2 (4%)	2 (4%)	0 (0%)
Postoperative	Child A	34 (72%)	20 (43%)	9 (19%)	5 (11%)	0 (0%)	0 (0%)
	Child B	13 (28%)*	0 (0%)	5 (11%)	3 (6%)	1 (2%)	4 (9%)

*P > 0.05.

### Follow-up and survival

After six months of postoperative follow-up, one patient died of systemic organ failure at the end of the follow-up period, and the remaining patients had not reached the OS endpoint.

### Observation and management of postoperative adverse effects

#### Fever

After interventional treatment, the necrotic tumor tissue is absorbed by the body, resulting in fever, which can occur 6 hours after surgery. Body temperature can reach up to 39°C, with symptoms lasting 3–4 days but usually subsiding within 1–3 days. In this study group, 37 patients (78.7%) developed a fever with a high body temperature (30).

#### Pain

In the process of interventional treatment, the liver tumor lesions caused by liver ischemia could stimulate the liver capsule, which would make the patient feel high pain. This may aggravate the patient’s concern about the disease and affect the patient’s confidence to fight it. In our study, 27 cases (57.4%) had muscle pain and pain at the puncture site. The pain score was 3–6 and was spontaneously relieved after 2–3 days through psychological care and relaxation therapy without any analgesics (31).

#### Bone marrow suppression

The patients were closely monitored for mucosal bleeding, ecchymosis, petechiae, hematuria, and bloody stools (32). Parameters such as the coagulation function, the blood routine, etc. were evaluated. Bone marrow suppression was performed by administering recombinant human granulocyte stimulating factor injection (100–200 µg) or recombinant human thrombopoietin (15000 IU) subcutaneous injection based on the clinician’s advice. Reactions such as fever, lower limb pain, rash, and fatigue, were closely monitored. Patients with severe myelosuppression should be protected to prevent cross-infection and receive blood transfusion care when necessary. In this study group, 23 patients (48.9%) had myelosuppression, including 14 leukopenia and 9 thrombocytopenia cases. After symptomatic treatment and care, the patients were able to complete the oncolytic virus injection combined with the standard treatment as planned (32).

#### Nausea and vomiting

Nausea and vomiting are caused by the toxic effect of chemotherapy drugs on the gastrointestinal mucosa during the interventional process (33). However, it only occurred in 5 patients (10.6%) within 24 hours of surgery, after which it gradually decreased and the symptoms were relieved or disappeared within 2 to 3 days. The color, smell, nature, and vomiting amount of the patient’s vomit were closely observed, and the presence of hematemesis or black stool was closely monitored to prevent complications such as gastrointestinal bleeding. For patients with severe vomiting, antiemetic treatments were prescribed, and they were advised to consume a light, digestible, and high-protein diet. For patients who cannot eat, the doctor’s advice has to be followed to strengthen fluid rehydration treatment, ensure normal liver and kidney perfusion, maintain liver and kidney function, and prevent liver and kidney damage. Preoperative and postoperative administration of antiemetic, stomach protection or H2 receptor antagonists was prescribed to prevent and treat gastrointestinal reactions.

## Discussion

Oncolytic viruses have been taking the front stage in biological therapy for cancer recently (34). Oncolytic virotherapy, a revolutionary tool for cancer treatment has shown promising results for the last two decades. More than a century ago it was first observed that cancer patients underwent cancer regression if they were infected with certain viruses. Revolutions in recombinant DNA technology have provided important tools to study the biology of viruses, thereby advancing biological therapy for cancer, resulting in the new generation of cancer therapeutics. While chemo and radiation therapies continue to be the chosen cancer treatment options, the serious side effects are a major drawback of these therapies. Intratumoral injection is the most commonly used method of administration of oncolytic viruses (35, 36).

Intratumoral injection is the most commonly used method of administration of oncolytic viruses, with safety evidence available from several phase III clinical trials, albeit insufficient. Intratumoral injections are complex to perform and are limited to tumors that are accessible through clinical palpation or direct imaging (8, 37). On the other hand, intravenous (IV) injection of the oncolytic virus is relatively rare and inefficient. IV-administered oncolytic viruses get diluted in the blood and suffer from serum antibody neutralization, thereby decreasing their bioavailability in the tumor tissue. IV administration may also cause systemic spread, causing a serious infection. A previous meta-analysis showed that intratumoral administration showed a significant improvement in efficacy (P = 0.0002) without IV administration (P = 0.99) (38).

The combination of an oncolytic virus with chemotherapy (27), radiotherapy, and targeted therapy, especially immunotherapy, can improve treatment efficacy. However, the systemic and physical barriers in the tumor microenvironment remain major obstacles to the clinical efficacy of oncolytic viruses, which need to be understood comprehensively (39). More patients may benefit from oncolytic viral drug therapy in the future. Local administration of the oncolytic virus is generally well tolerated, and the most common adverse reactions are influenza-like symptoms and local reactions at the injection site. Among them, influenza-like symptoms are often manifested as elevated body temperature, myalgia, fatigue, nausea, vomiting, headache, etc. Generally, they will be relieved without any treatment after the virus infusion. Some patients with intolerance or elevated body temperature can return to normal after receiving symptomatic treatment and care. Local reactions are often manifested as pain, rash, erythema, peripheral edema, etc., and most reactions subside (40).

## Conclusions

In this study, 47 patients received an intratumoral injection of an oncolytic virus combining chemotherapy, targeted therapy, and immunotherapy (41). As an emerging tumor immunotherapy method, the oncolytic virus has been approved by the regulatory authorities in China and many European and American countries. Oncolytic viruses kill cancer cells by multiple mechanisms. A large number of clinical studies have confirmed that it has a good clinical benefit and a safe use record, and its infection of tumor cells has enhanced the anti-tumor immune response and can produce a relatively lasting response (35). In this study, 47 patients completed 2 courses of oncolytic virus injection, and the lesions in the target organs were significantly reduced. The main complications of oncolytic virus injection in the patients in this study were postoperative fever, pain, vomiting, myelosuppression, etc. Comprehensive nursing intervention can meet the needs of patients after oncolytic virus injection, and provide effective care for patients who suffer postoperative complications. Apart from the therapeutic interventions, family members, appropriate health knowledge, and psychological support can effectively improve mood and cognition (1), thereby potentially improving clinical outcomes (6).Biological therapy for cancer, although relatively complex and challenging, is the preferred treatment option owing to good efficacy, limited side effects and being less painful to cancer patients. So far clinical trials report no deaths or clinically serious adverse events attributed to oncolytic virotherapy. In cancer treatment the patient’s safety is of utmost importance and treatment, using oncolytic viruses seems to be the most promising in this aspect. Most of the oncolytic viruses chosen for cancer therapy are attenuated strains or strains that can infect and replicate in humans without causing any serious disease. It is also important that the viruses chosen must be capable of utilizing the host immune system to recognize and destroy the cancer cells (11).

## Data availability statement

The original contributions presented in the study are included in the article/supplementary material. Further inquiries can be directed to the corresponding authors.

## Ethics statement

The studies involving human participants were reviewed and approved by the Ethical Investigation Committee of Ruijin Hospital affiliated with the School of Medicine, Shanghai Jiaotong University, China. Ethical approval code: (2020). Written informed consent was obtained from each patient in the form of a questionnaire for the collection and analysis of applicable clinicaldata. The patients/participants provided their written informed consent to participate in this study.

## Author contributions

JiZ, QH and MW, LH contributed in the study design, data collection, data analysis, data interpretation, literature search, and writing of the article. JiZ contributed in the literature search and writing of the article. JiZ, QH, DM contributed in the data collection, analysis, and interpretation. JiZ, MW, and DM contributed in the data analysis and data interpretation. JuZ and MW contributed in the study design and data interpretation. All authors contributed to the article and approved the submitted version.
